# The Kokel of Southern Siberia: New data on a post-Xiongnu material culture

**DOI:** 10.1371/journal.pone.0254545

**Published:** 2021-07-16

**Authors:** Timur Sadykov, Gino Caspari, Jegor Blochin, Sandra Lösch, Yulija Kapinus, Marco Milella

**Affiliations:** 1 Institute for the History of Material Culture, Russian Academy of Sciences, St. Petersburg, Russia; 2 Department of Archaeology, University of Sydney, Sydney, Australia; 3 Institute of Archaeological Sciences, University of Bern, Bern, Switzerland; 4 Department of Physical Anthropology, Institute of Forensic Medicine, University of Bern, Bern, Switzerland; 5 Volga-Ural Center for Paleoanthropological Research, SSSPU, Samara, Russia; University at Buffalo - The State University of New York, UNITED STATES

## Abstract

From the end of the Xiongnu Empire to the establishment of the first Turkic Khaganate, the territory of Southern Siberia sees the emergence of distinctive local material cultures. The Kokel culture is essentially unknown in the international English-language literature even though archaeological sites pertaining to this material culture are among the most common in Tuva (Southern Siberia). This makes them important for the understanding aspects of the sociocultural dynamics following the collapse of the first “steppe empire”. Here we present the results of the study of a Kokel funerary site recently excavated near the Early Iron Age kurgan Tunnug 1 and discuss the data in the context of the available Soviet and Russian literature. The Kokel culture substantially differs from the material culture of the Xiongnu and has to be seen as a largely independent cultural entity of small tribal groups without a pronounced social hierarchy engaging in frequent violent local conflict.

## 1. Introduction

The time between 2^nd^ century BCE and 5^th^ century CE in Central Asia is traditionally referred to in Soviet archaeological literature as the “Hunno-Sarmatian Period". This broad period is fuzzy and consists of a plethora of local cultural phenomena. The Hunno-Sarmatian Period shows clear differences with both the preceding Early Iron Age, and the subsequent medieval material cultures but is comparatively heterogeneous internally. This is why subdivisions have been made to address local questions more properly. Most geographical regions of Central Asia and Southern Siberia have been assigned a single “culture” within a traditional culture history framework that identifies based on assemblages of material remains. These cultures usually cover the entire temporal range of the Hunno-Sarmatian period. The subdivision of archaeological cultures into chronological subunits remains a mainstay of post-Soviet archaeology. Some geographic areas, however, exhibit changes in the composition of assemblages of material remains which merit the creation of additional cultural sub-units in order to understand socio-economic dynamics at a higher resolution. One such area is the mountainous region south of the Sayan Mountains, Tuva Republic.

**Fig 1:**
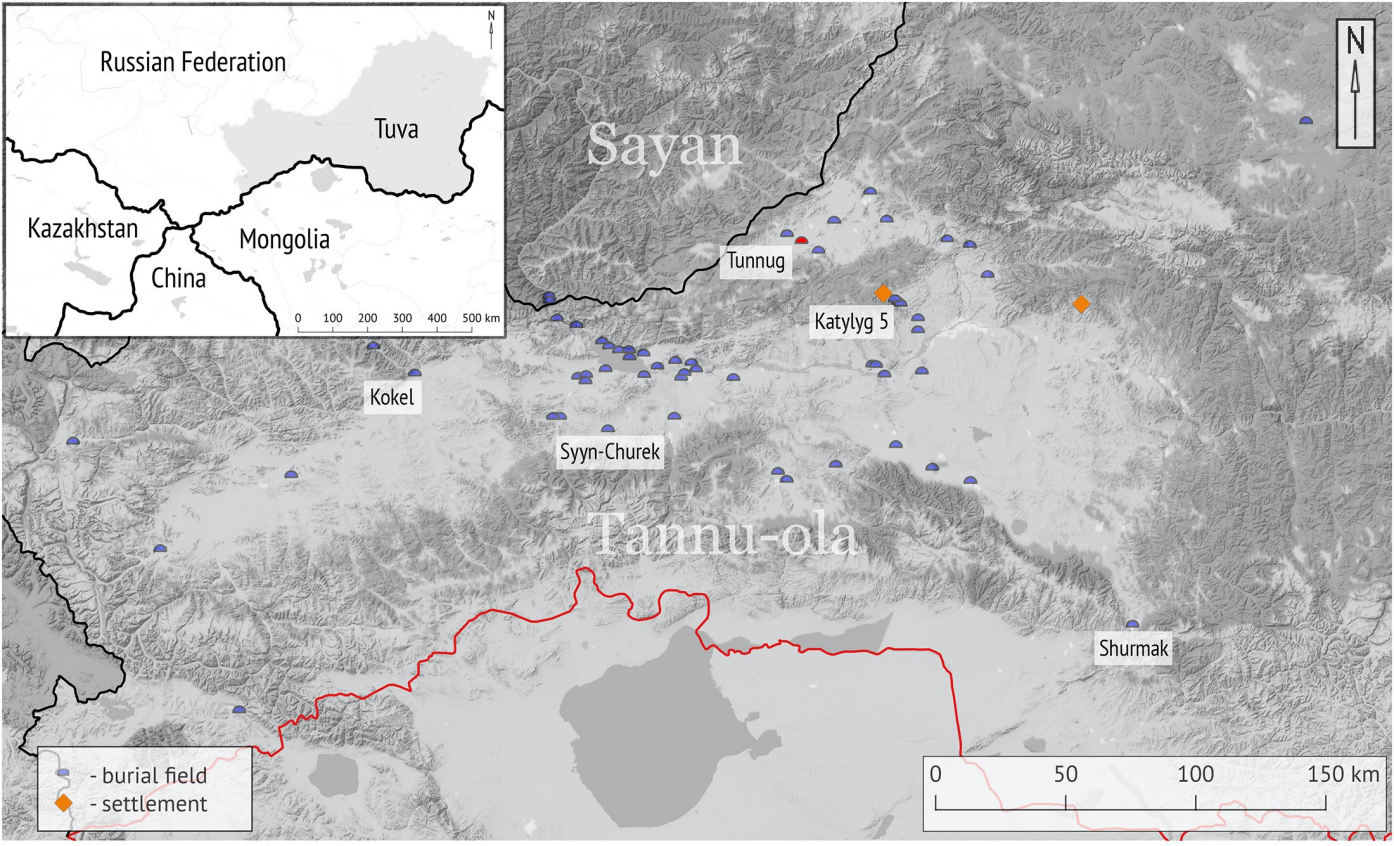
Map of investigated Kokel archaeological sites with Tunnug marked in red. Based on [9]. The red line marks the modern border between the Russian Federation and Mongolia. We acknowledge the use of open access data as a basemap downloaded through Stamen Design under (CC BY 3.0) license. The map was created in QGIS 3.10.

First sites of the Kokel culture were discovered in 1915–16 by A.V. Adrianov and a decade later (1926–1929) by S. A. Teploukhov, but the results of these excavations remained unpublished [1]. It was only in 1958 when S.I. Weinstein and L.R. Kyzlasov attempted a definition and classification of the archaeological materials. These researchers independently named the same assemblage of materials *Syyn-Churek* culture [2] (based on the name of the excavated site, cf. Fig 1) and *Shurmak* culture [3] (also based on the site name cf. Fig 1). Stylistic comparisons classified the Kokel culture to belong within the chronological borders of the Hunno-Sarmatian period (2^nd^ century BCE–5^th^ century CE). Due to a limited amount of archaeological research that had been conducted in the area and in the absence of radiocarbon dating, this material culture complex filled the gap between the Early Iron Age and the Medieval period and was therefore seen as stretching from start to the end of the Hunno-Sarmatian period. The defining features of these assemblages were described as a superimposition of allochthonous (Xiongnu) cultural elements on the local post-Scythian tradition. D. G. Savinov coined the current term *Kokel* culture more than two decades later [4].

The rough chronological frame assigned to the Kokel culture had to be substantially revised following more extensive archaeological research being conducted in Tuva [1: 18–28]. In particular, the redating of the Late Scythian sites to the 2^nd^ / 1^st^ century BCE, based on stylistic analogies with the well-defined material culture of the Xiongnu [5], prompted a shortening of the time span associated with Kokel. The Bai-Dag 2 site features several terrace tombs related directly to the Xiongnu [6]. Newly discovered sites in Tuva dating to the first centuries BCE exhibit connections with the material culture of the Xiongnu, but do not show any similarities with the Kokel culture [7]. With a general lack of radiocarbon dates, comparative methodologies suggest that none of the Kokel (or “late Shurmak” [8:748]) sites appear before the first century CE [1, 6, 8, 9]. Additionally, there is no obvious connection between the Kokel culture and the Xiongnu material culture. No archaeological sites show a mixture of items from the two material cultures.

The concept of the "Kokel archaeological culture" does have a somewhat confusing history. In 1992, Mandelstam and Stambulnik divided the Hunno-Sarmatian time into an early and a later stage which included the Kokel culture [6]. They relied on the two sites Bai-Dag 2 and Aimyrlyg 31. The archaeological materials of these sites have been published in sum without a proper chronological separation. Given the heterogeneity of the materials, the sites very likely had a longer run-time and included materials from different usually clearly separated chronological contexts. This is where some of the confusion stems from. H. Parzinger [8] then divided the “Shurmak culture” into an early (Aimyrlyg) and a late (Kokel) stage. He correctly determined the date of the “Kokel stage of the Shurmak culture” to lie in between the 2^nd^ and 4^th^ century CE. However, overall the “Shurmak culture” was presented as a single phenomenon which is problematic. The term “Aimyrlyg stage” cannot be properly used or verified since the materials from this site have not been published and also do represent a chronologically mixed context. M. Kilunovskaya and P. Leus have recently excavated sites that very clearly date to the 2^nd^ and 1^st^ centuries BC possibly extending into the 1^st^ century CE [7]. They determine a separate “Ulug-Khem culture” in Tuva which is lacking direct correspondences with the “Kokel culture”. The previously proposed Kokel stage of the Shurmak culture is based on partially mixed and unavailable archaeological materials. We show in our paper that a clear separation of the Kokel culture from previous archaeological contexts is possible if we base our analysis on the published materials and radiocarbon dates. In our opinion it is important to show that the Kokel culture is a separate phenomenon. We would like to avoid reintroducing confusing mixed sites and terms that do not accurately reflect the material record. These practices would only serve confusion and a perpetuation of categories that have been shown not to be reflective of the data we gather in the field. The conceptual separation of the Kokel culture as a distinctive phenomenon is an essential outcome of this research.

Research in the last decade, including our own, has further enhanced the categorization of archaeological materials from this period and narrowed down the chronological range [9: 98]. The spread of data and interpretations beyond a Russian community of archaeologists remains, however, limited. The goal of this paper therefore is to properly introduce this archaeological culture, provide substantial new data, and contextualize it in order to fill the gap in the international literature.

### 1.1 Limitations and accessibility of literature

The archaeological literature on the Kokel culture is scant. This can be attributed to a lack of aimed research projects focused on this material culture. The majority of Kokel finds were either identified during—rarely published—rescue excavations or were simply a by-product of research focused on other time periods. Only a few dedicated published studies can be noted:

Between1960 and 1970 three volumes of the «Proceedings of the Tuva Complex Archaeological and Ethnographic Expedition» [10–12] presented a comprehensive review of the archaeological finds from the eponymous Kokel burial field and several smaller sites. The funerary site of Kokel included 446 individuals distributed among nine burial mounds and 41 archaeological structures. Up to now, it is still the best-known archaeological site of this culture. Kenk [13], whose work is, to date, the only major publication on the Kokel culture in a non-Russian language, later revisited these data. While many of Kenk’s conclusions, especially concerning the periodization and chronological assumptions are outdated, all data generated after his publications were published exclusively in Russia and therefore remain out of reach for most international scholars.

Kyzlasov [14] published the results of ten years of excavations in Tuva as a monograph where, in addition to the results of his research, he also reported archival data from earlier, unpublished, research by Adrianov and Teploukhov. Kyzlasov [14] subdivided all considered sites into “Early Shurmak” (2^nd^ BCE–1^st^ CE) and “Late Shurmak” (2^nd^ c. CE–5^th^ c. CE) period.

From 1965–1980 a rescue excavation project documented sites in the flood zone of the Sayano-Shushenskaya hydroelectric power plant in Central Tuva. Here, more than 40 sites with remains belonging to the Kokel material culture were excavated. These results were first published in a summary form that aggregated data [6], which was then followed by a more detailed report published in 2010 [15]. To date this is the most comprehensive research conducted on the Kokel culture since the excavation of the eponymous site.

From 2015–2018 articles devoted to the first known settlement of the Kokel culture—Katylyg 5—were published [16, 17]. The discovery of previously unknown iron metallurgy furnaces, a fortification system, and new types of ceramics, called for a revision of previous theories about the origin and development of this culture.

### 1.2 Geographic distribution of the Kokel culture

The geographical distribution of the Kokel culture is so far limited to the territory of the Republic of Tuva (Fig 1). The Northern and Western limits of the Kokel cultural landscape are represented by the Tashtyk culture [18] in Khakassia and the Minusinsk Territory, and the Bulan-Koba culture [19] in the Altai Republic. These different material cultures are separable spatially, hinting at limited long-distance contacts at the time. Towards the south in northwestern Mongolia, this period has been rarely investigated. Judging by the absence of even incidental finds along the border of southern Tuva, the distribution of Kokel sites might be limited to the Sayan Mountains north of the Tannu-Ola ridge.

### 1.3 Biological and material culture characteristics

Three categories of Kokel archaeological sites can be distinguished—funerary contexts, ritual structures, and settlements. Note that, although most of late prehistoric archaeology on the steppe is focused on the analysis of funerary assemblages, recent investigations have started to incorporate the study of other contexts. For the Kokel culture these inquiries are, however, in their infancy with only one settlement excavated (cf. Fig 1, Katylyg 5) and another potential settlement site identified.

#### 1.3.1 Funerary contexts and anthropological patterns

Kokel burials are mainly represented by inhumations, each one covered by a funerary mound. These low mounds tend to coalesce over time, leading to amorphous structures counting in some cases more than a hundred burials. In western Tuva, such complexes have been studied at Kokel and recorded during surveys (1: 27). Individuals are usually interred within wooden coffins inside simple pits which are in rare cases outlined with stones. Burials without coffins are also known. Although burials tend to vary in their orientation, there is a tendency for the deceased to be oriented with the head in the northwest and the feet in the southeast [1, 9]. But this is not a consistent pattern with a large number of exceptions.

The burial mounds of the Kokel culture lack standardized structural elements with their outline being alternatively circular, semi-oval or amorphous. It can be noticed that the larger burial mounds are not planned complexes, but rather the result of the diachronic accumulation of burials. Consequently, the burials can be found both under and between the stone layers composing the mound itself. They are not necessarily placed in the centre of the mound, as this is the case with other mound building archaeological cultures on the Eurasian steppes. In addition to constructing their own mounds, Kokel burials are found as implants in funerary monuments of other earlier steppe cultures like Early Iron Age burial mounds (1: 19, 9: 101–103).

**Fig 2:**
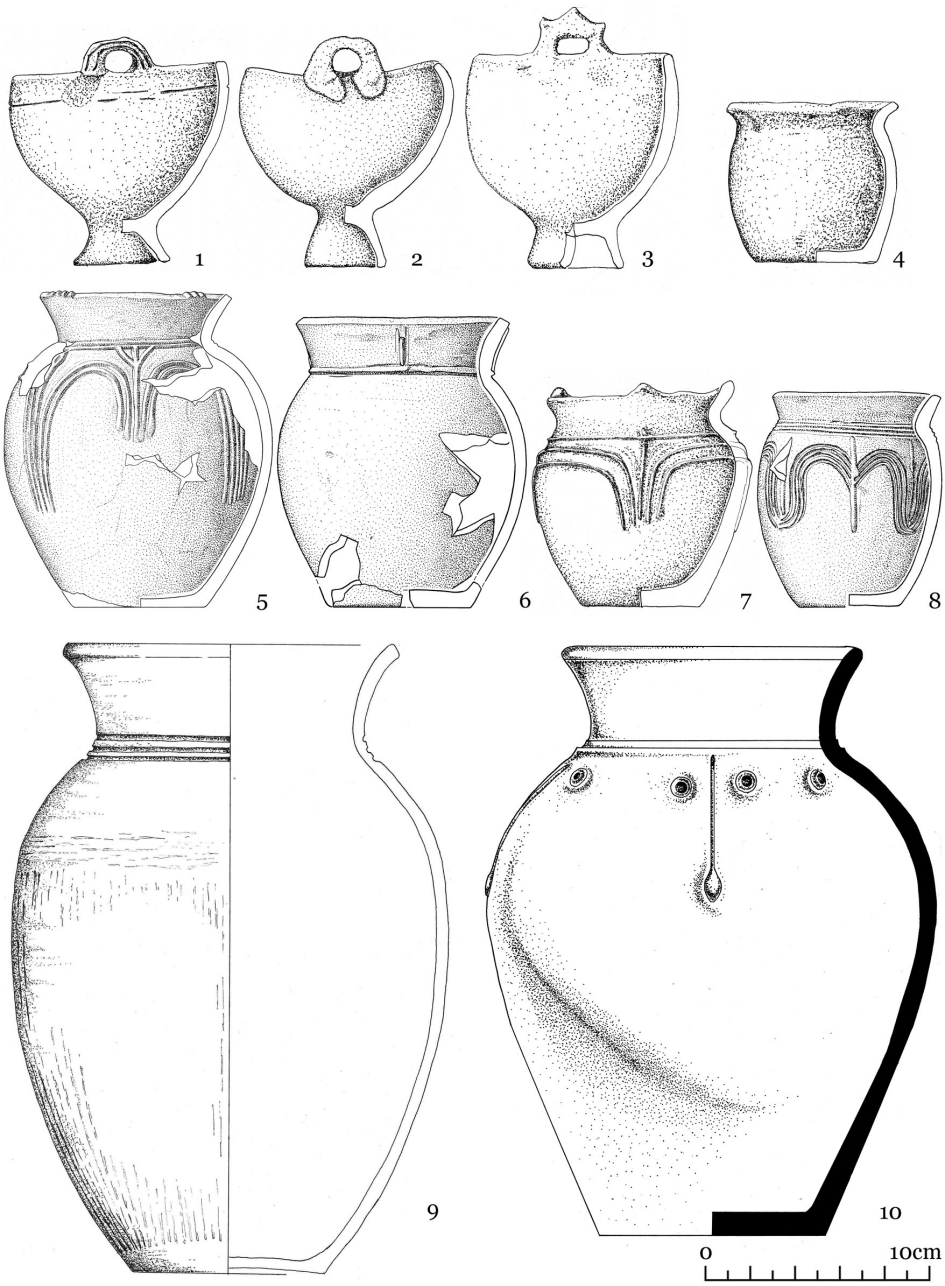
The most frequently found vessels. Diagnostic ceramics of the Kokel archaeological culture include cauldron-shaped vessels (1–3) and vases of different sizes (4–10).

Kokel burials usually contain ceramics (one or two vessels) (Fig 2), a variable number of iron knives and buckles, and animal bones related to meat offerings (9: 98–99). Household items and weapons are occasionally found. Among the latter, arrowheads are the most frequent, whereas bow linings and backswords are less common. There is, however, a bias due to preservation influences as the exceptionally preserved burials at the Kokel burial ground showed, where items made of wood and birch bark were recovered. Many grave goods are not full-sized items but rather miniature models. Bronze itemss are overall very rare.

Previously, anthropological data on Kokel populations were available only for the eponymous site [13:144–165]. These data include more than 380 skeletons from different mounds. The demographic distribution is characterized by a mortality peak between ca. 20 and 50 years of age and an overrepresentation of males vs. females (respectively 66.2% and 33.8% of anthropologically sexed skeletons). The latter bias was interpreted by Kenk [13:93] as the possible result of infanticide targeting female offspring. According to Kenk, the combined anthropological and archaeological data from Kokel indicate a social differentiation built around sex and age as dominant axes. Features like grave depth and the distribution of gold items suggest are seen as indicative of social status. Shallow burials mostly include remains of subadults and adult females, whereas deeper ones at more than 2m of depth almost exclusively feature adult males. Burials including gold items include mostly males between 40 and 60 years of age.

#### 1.3.2 Ritual structures

Ritual structures, usually referred to as “memorial complexes” [20], are one of the most frequently occurring type of feature in the Kokel archaeological record. We prefer to use the term “over-vessel mound” for these structures, which avoids the interpretive aspect. These stone mounds can be small, up to 3m in diameter, or large of comparable size to the funerary mounds. Their central and usually only artefact is a ceramic vessel placed inside a cluster of stones. In some cases, several vessels, separately placed, can be found within one mound (20: *272*; 15: *31*, *33*, *39 etc*.). Rarely, burials were discovered under such mounds near the edge of the feature, with the vessel in the central position [15: *36*, *76*]. This suggests an association of the over-vessel mounds with funerary rituals. Although over-vessel mounds are usually found together with burial mounds, there is no unambiguous planigraphic correspondence between them. Sites consisting only of over-vessel mounds are known [20]. In most cases, Kokel burials and over-vessel mounds are located in close proximity to older burial mounds, for example from the Early Iron Age. Over-vessel mounds often include traces of fires, ashes, and fragments of ceramics and therefore they are suggested to be associated with ritual activities involving fire. In addition, Kokel vessels are also found as implants in funerary monuments of earlier steppe cultures (9: 99).

#### 1.3.3 Settlements

**Fig 3:**
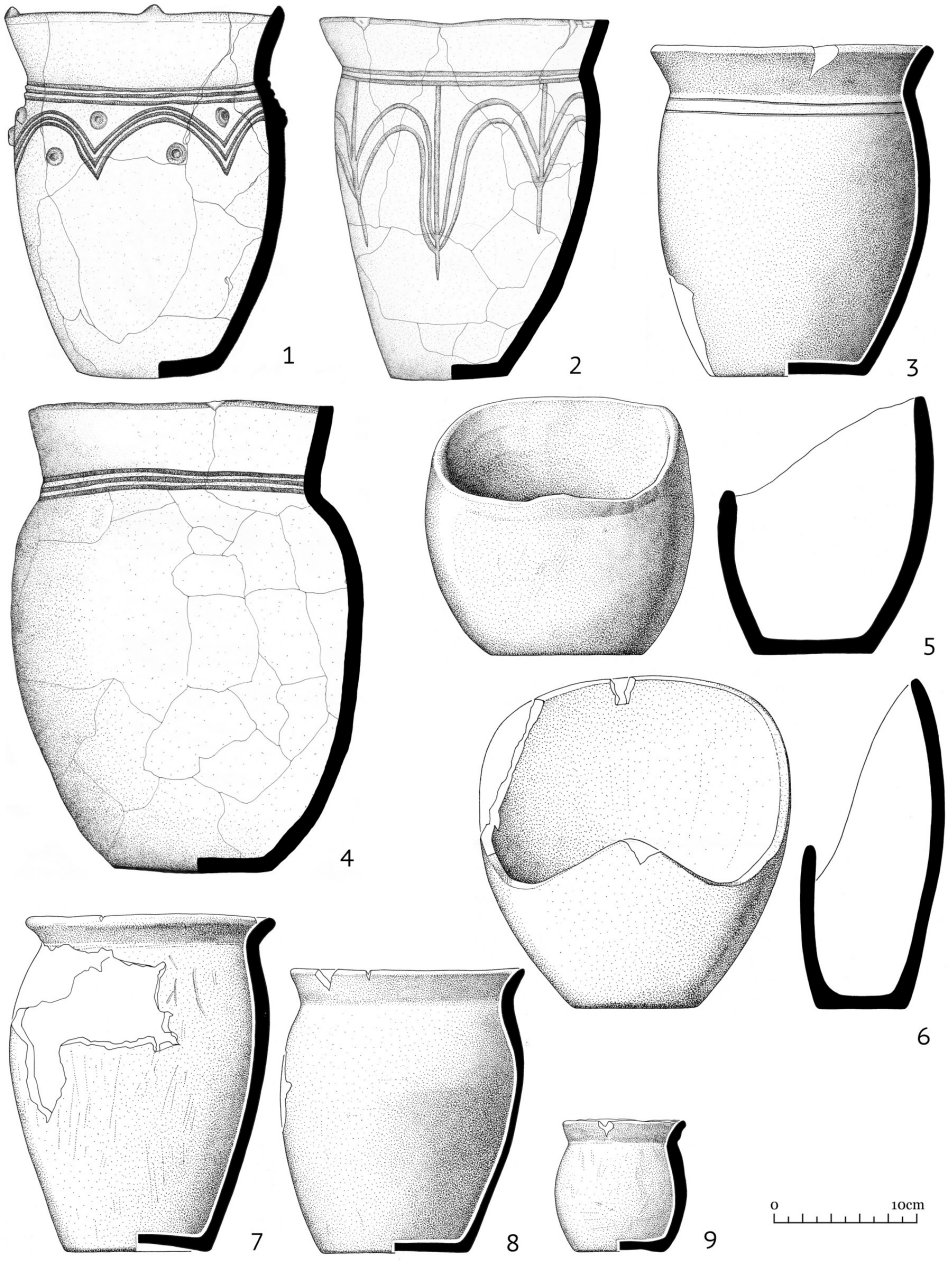
Kokel ceramic forms recovered from the Katylyg 5 settlement. 1–4 and 7–9 vase forms, 5–6 vessel with asymmetric rim.

From 2014–2015, the fortified settlement Katylyg 5 was excavated by one of the authors [16, 17]. Another settlement—Chyvarlyg 1 is known from surveys and can tentatively be attributed to the same time period [21]. The same vase forms found in funerary contexts are also represented within settlements (Fig 3). Cauldron-shaped vessels have so far not been found in a settlement context and it is possible that this type of vessel was made specifically for burials. An unusual type of vessel with an asymmetric rim found solely in settlements, is unique for Inner Asia and has yet to be associated with a specific purpose (Fig 3: 5,6) [16: *80*].

Katylyg 5 showed that the populations associated with this material culture led a semi-nomadic life in relatively small groups. The settlement area of around 4200 m^2^ is estimated to have provided space for up to 30 inhabitants. Based on the animal bones, the composition of herds is relatively close to what can be observed today among Tuvinian pastoralists. Currently, this area is used as a summer pasture. In autumn, nomads descend from the taiga to the steppe-covered foothills of the mountains to allow for winter grazing. Apart from ethnographic parallels for usage patterns, there were no traces of substantial dwellings found in Katylyg 5. This might suggests that light structures have been used, that do not provide the necessary protection during harsh winters in the Taiga. Livestock consisted mainly of ovids, caprids, bovids and some equids. Domestic animal food sources were supplemented through hunting of cervidae, suidae, and leporidae [17: 140].

Iron was the main material for tools and weapons, whereas bronze was absent. Smelting furnaces are fired with charcoal from local larch forests. Fortifications of even a seasonal (summer) settlement in the high-mountain taiga zone indicates an environment characterized by violent conflict. However, no destruction of the Katylyg 5 site was noted during its excavation [17].

### 1.4 The Tunnug 1 project

**Fig 4:**
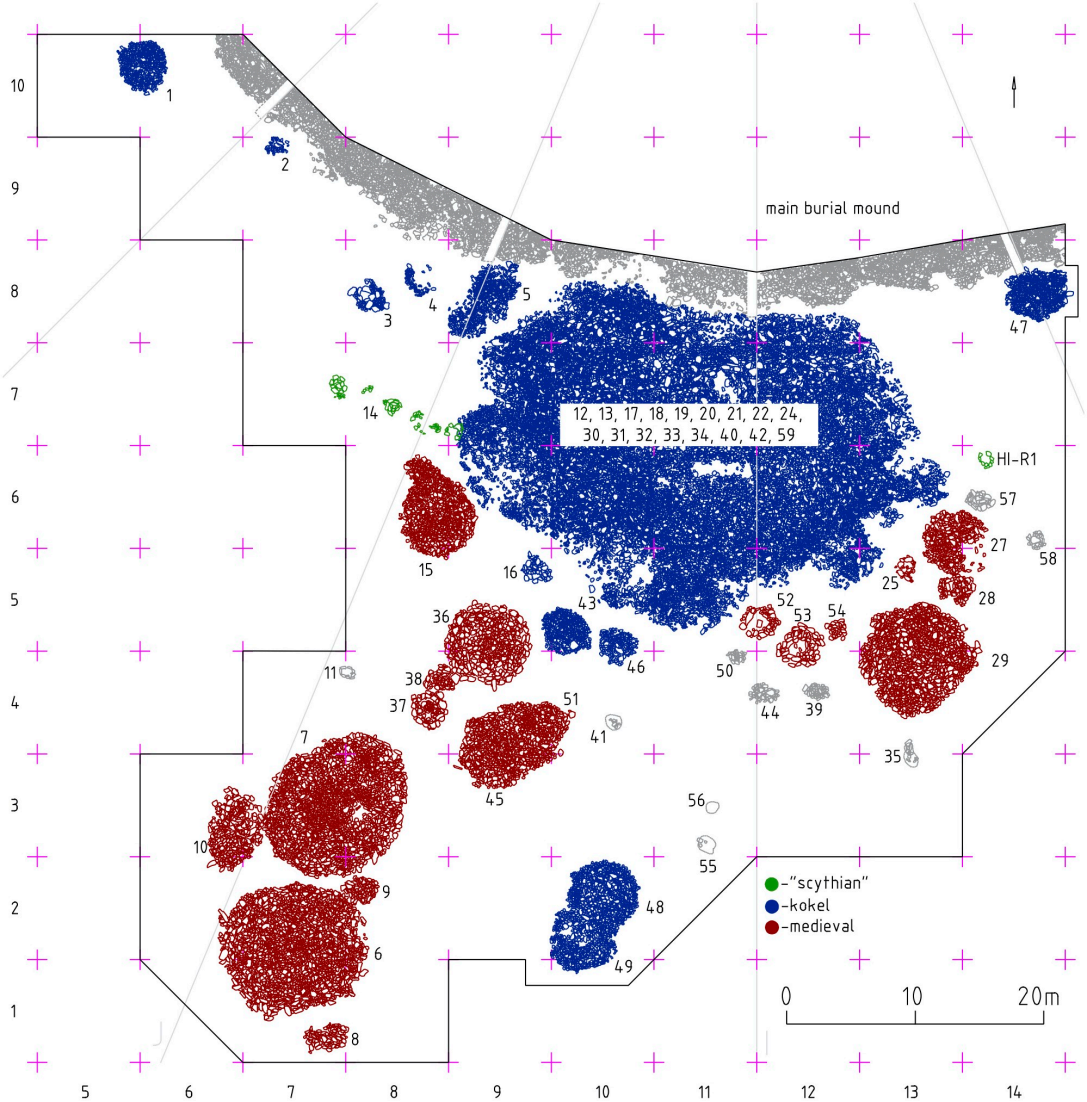
Planigraphy of the southern periphery of Tunnug 1. The main burial mound dates to the 9th century BCE [23] and formed the nucleus for later funerary and other ritual activities. Numbers denote individual archaeologically separable structures.

The Uyuk Valley, or “Valley of the Kings” in Tuva Republic is mainly known for its large burial mounds and the Early Iron Age cultural heritage [22]. The interest in the Bronze Age Iron Age transition and the emergence of highly mobile nomadic pastoralism in the eastern Eurasian steppes in conjunction with monumental burial mounds and a steep hierarchical organization of nomadic societies led to the establishment of the Tunnug 1 excavation project [23]. Our first excavations in 2018 showed that the use of the site for funerary purposes covered more than two millennia, including an intensive usage of the southern periphery of the main burial mound during the Kokel period [24]. In 2019, a geophysical surveys led to the discovery of an extensive periphery of the Early Iron Age burial mound [25]. From 2018 to 2019 we investigated most of the stone structures which we could assign to the Kokel culture (Fig 4). Among them were both smaller over-vessel mounds as well as an amorphous stone mound with a maximum diameter of 28m covering dozens of burials and apparently had been used over an extended period.

## 2. Methods

### 2.1 Archaeological excavation

Field research was conducted under licenses No. 0434–2018 and No. 0590–2019 issued to T.Sadykov from the Institute for the History of Material Culture, Russian Academy of Sciences. The permits were issued by the Russian Ministry of Culture. The topographic conditions and the fact the site is situated in a floodplain with seasonal inundation, the complex structure of the site, and the area extent of several thousand square meters required a specific excavation protocol. The excavation area of the burial site was sectioned with a box grid into quadrants of 8x8 m. We located the conventional zero in the south-western corner of the excavation area. Lines of the squares are numbered from 1 to 25 on the horizontal axis [X] and from 1 to 24 on the vertical axis [Y]. In accordance with the requirements of Russian archaeological authorities, each individual “object”—which denotes an entire separable archaeological structure—receives its own stratigraphic profile and a plan of each archaeological layer inside. These established rules as well as local research traditions do not allow a strict execution of a stratigraphic excavation. In most cases, the approach is comparable to the excavation of individual stratigraphic units, but deviations can occur. During excavation, most archaeological objects were cut with a real profile. However, in cases where we could not obtain a real profile in the field, we excavated the objects by documenting plans of thin conventional horizons. We then reconstruct the stratigraphic profile during post-processing. The reason for this approach lies in the instability of pit fillings, which can consist of an unstable mixture of small and large stones with loose sandy loam. The groundwater leads to instability of the real stratigraphic profile and could result in a collapse before the documentation is completed. The documentation of each stone layer by means of photogrammetry and 3D-modeling allowed us to subsequently reconstruct the exact 3D location of each stone in the filling and thus create an adequate picture of the stratigraphy.

Every cleaned layer was documented using 3D modelling of the excavated areas by means of a structure from motion approach. We referenced all documentations in the coordinate system created during the 2017 survey [23]. After each stage of the excavation, we documented every unit with UAVs and digital photography. From these sets of photographs, 3D-models, elevation models and orthophotographs of every unit were generated using the Agisoft Metashape software. We created object drawings in Autodesk AutoCAD using data from the 3D-models. All artefacts (animal bones, ceramics fragments, iron, bronze, bone, stone items), as well as wood, soil or bone samples taken for analysis were recorded using a total station in the coordinate system adopted for the excavation.

All necessary permits were obtained for the described study, which complied with all relevant regulations. The 2018 excavation campaign at Tunnug 1 was carried out under OL No. 434 dated May 16, 2018 (Ministry of Culture of the Russian Federation). The 2019 excavation campaign at Tunnug 1 was carried out under OL No. 0590–2019 dated May 31, 2019 (Ministry of Culture of the Russian Federation).

### 2.2 Age-at-death estimation and sex determination

Subadult age-at-death was estimated based on the development and eruption of deciduous and permanent teeth, measurements of long bone diaphysis, and degrees of epiphyseal fusion [26–29]. We estimated age-at-death of adult individuals on the basis of the morphological changes of the auricular surface of the ilium, pubic symphysis, and sternal ends of the ribs [30–32]. For the aim of analysis, individuals were then grouped in age classes: neonates (up to 4 months of age), infants (4 months-3 years), children (3–12 years old), adolescents (13–18 years old), young adults (19–35 years old), middle adults (35–50 years old), and old adults (≥50 years old). Sex was estimated only for adult individuals based on the dimorphic features of the cranium, mandible and innominate bone [33–35].

## 3. Results

### 3.1 Planigraphy

**Fig 5:**
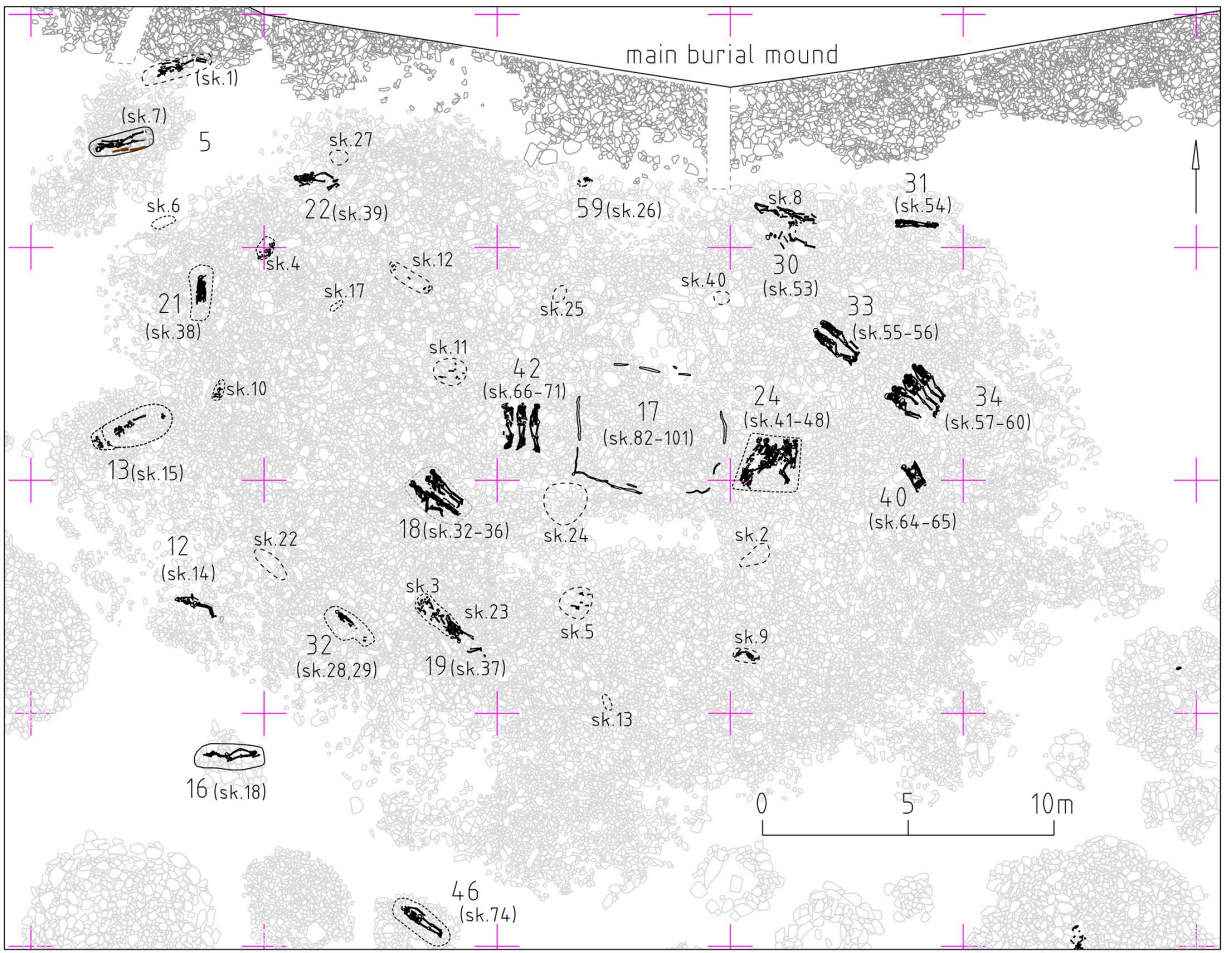
The large amorphous Kokel burial mound in the southern periphery of the Tunnug 1 site. It includes individuals buried in pits as well as individuals only minimally covered with stones. Larger numbers denote individual archaeologically separable structures belonging to the amorphous Kokel mound (5 is a separate smaller feature), small sk.-numbers denote skeleton numbers.

In the study of the southern periphery of the Early Scythian mound Tunnug 1, we excavated a large burial mound, several separate mounds and ritual structures pertaining to the Kokel archaeological culture (Fig 4). The Kokel burial mound can be stratigraphically separated into 18 archaeological structures (Fig 5). These are not simultaneous, but were rather progressively added resulting in an increase in size of the mound.

An object (large numbers in Fig 5) describes a separate structural unit as part of a mound, usually a pit with one or more inhumations. In addition, we found individual skeletons (sk. numbers in Fig 5) interred within the stone layer of the burial mound. These burials were found in the stones of the amorphous mound without a pit or any other structural separation. In some cases, funerary equipment belonging to the same cultural tradition accompanied the burials, sometimes the individuals were interred without grave goods. In total, we recovered skeletal remains of 67 individuals. The remains of at least additional 20 individuals were documented in the central pit (object 17) which likely represented a multiple burial, possibly a mass grave disturbed by later anthropogenic activity.

### 3.2 Physical anthropology

**Fig 6:**
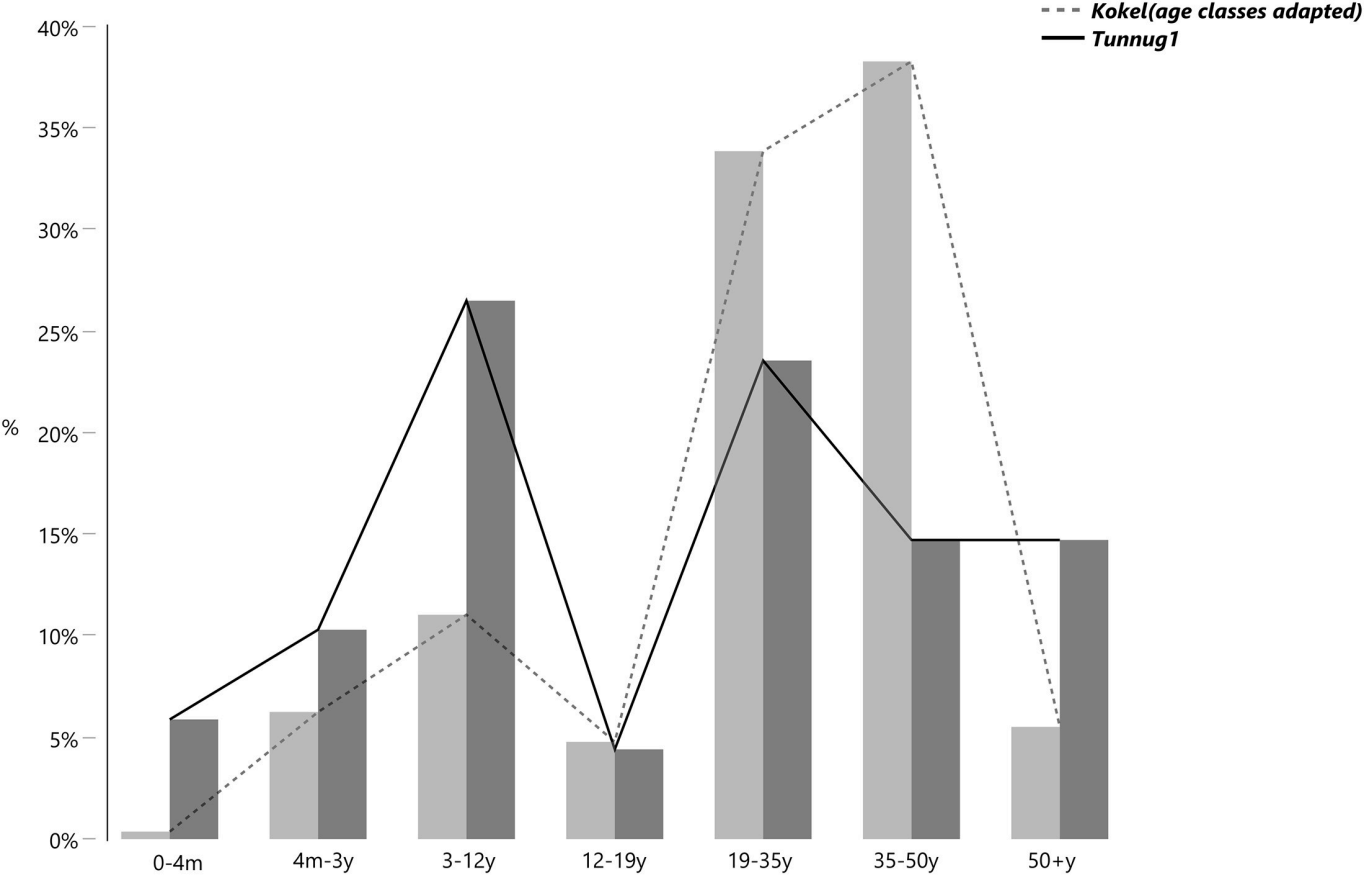
Age classes at Tunnug 1 (dark grey) and Kokel (light grey) in direct comparison.

Fig 6 illustrates the main demographic patterns using broad age classes, and its comparison with data from the site of Kokel [13:144–165]. In Tunnug 1 children (3–12 years old) and young adults (19–34 years old) are the most numerous age-at-death classes; among adults the frequency of males and females is 63.2% and 36.8% respectively. The sex ratio is therefore strikingly similar to that of Kokel, whereas the age distribution slightly different (Fig 6). A large amount of males at Tunnug 1 present perimortem trauma suggestive of combat and/or ritual practices [36], and this type of bias may be the source of bias in sex ratio observed. Perimortem trauma are also present at Kokel [37], and warfare may well be the source of the sex bias at this site.

### 3.3 Archaeological materials

Below we provide a detailed description of three objects selected to illustrate the variability of the Kokel material culture. Object 46 (skeleton 74, a male of 40–50 years of age) is a separate mound—the most typical burial form of this material culture. Object 33 (skeletons 55 and 56, two males between 30 and 40 years of age) is one of the central burial pits in the large amorphous mound. Object 22 (skeleton 39, a female aged 25–30) is a rich, and rather atypical burial.

#### 3.3.1 Object 46

**Fig 7:**
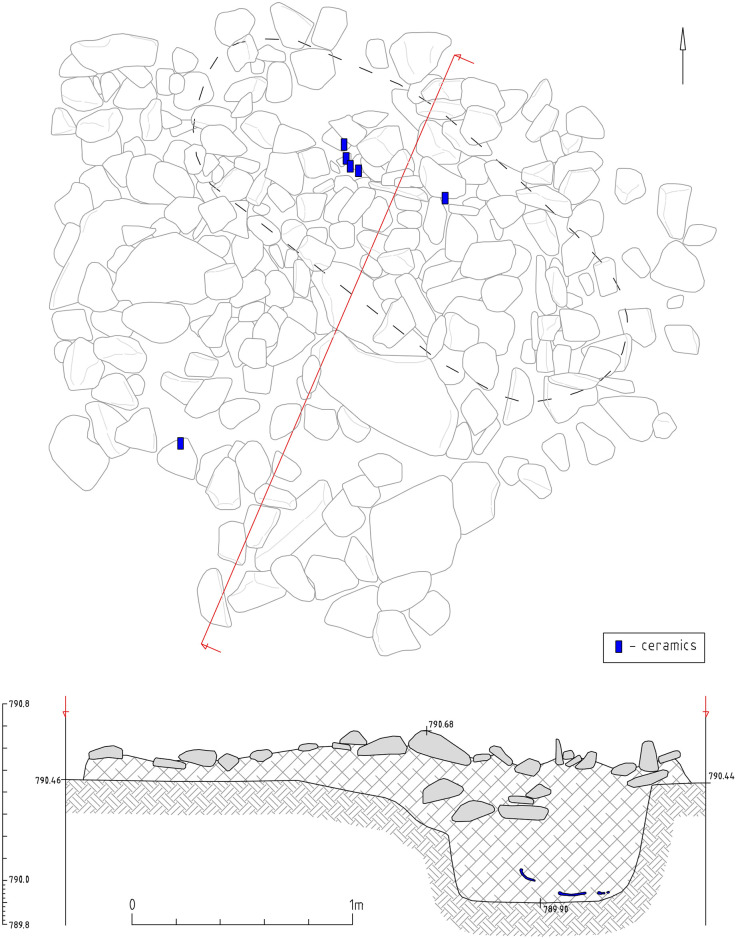
Object 46. Planigraphy and profile with the positions of individual ceramic shards indicated as blue rectangles.

**Fig 8:**
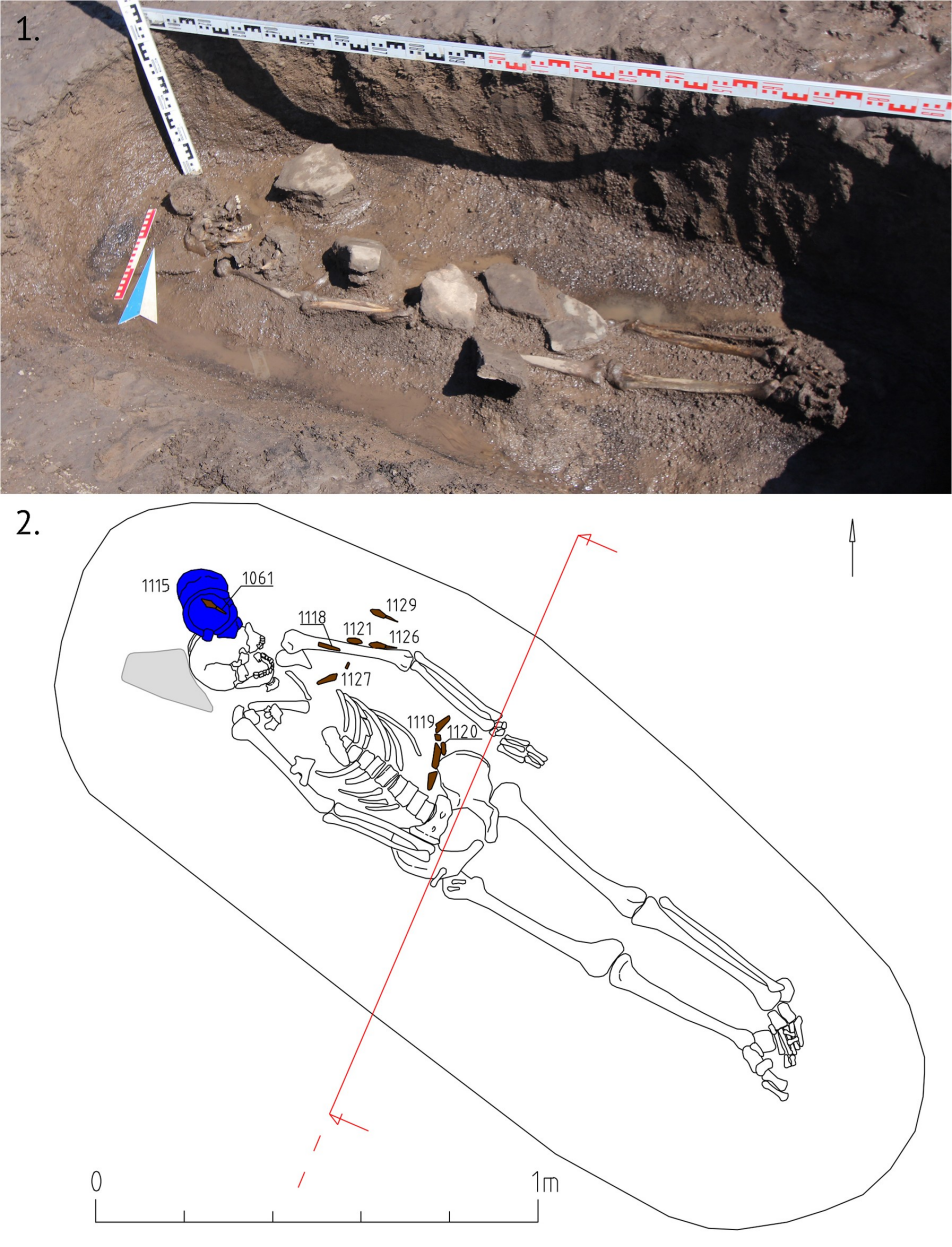
Object 46. A burial in supine position with the head towards the northwest, the body covered with larger stones. Numbers in the drawing correspond to illustrations in Figs 9 and 10.

Object 46 is a stone mound with a diameter of 3 m and composed of 2–3 stone layers. Fragments of a ceramic vessel were found between the stones belonging to the same type as the ceramic vessel retrieved from the burial beneath the surface). The burial pit is not located in the center of the mound (Fig 7). We found stones directly on the top of the skeleton, another characteristic of Kokel burials (Fig 8). Often, the filling of burial pits consists merely of stones. The burial pit measured 2.0 x 0.7m at a depth of 0.5m from the level of the ancient surface. Stretched out in a supine position with his head towards the northwest, the buried male was around 40–50 years of age.

**Fig 9:**
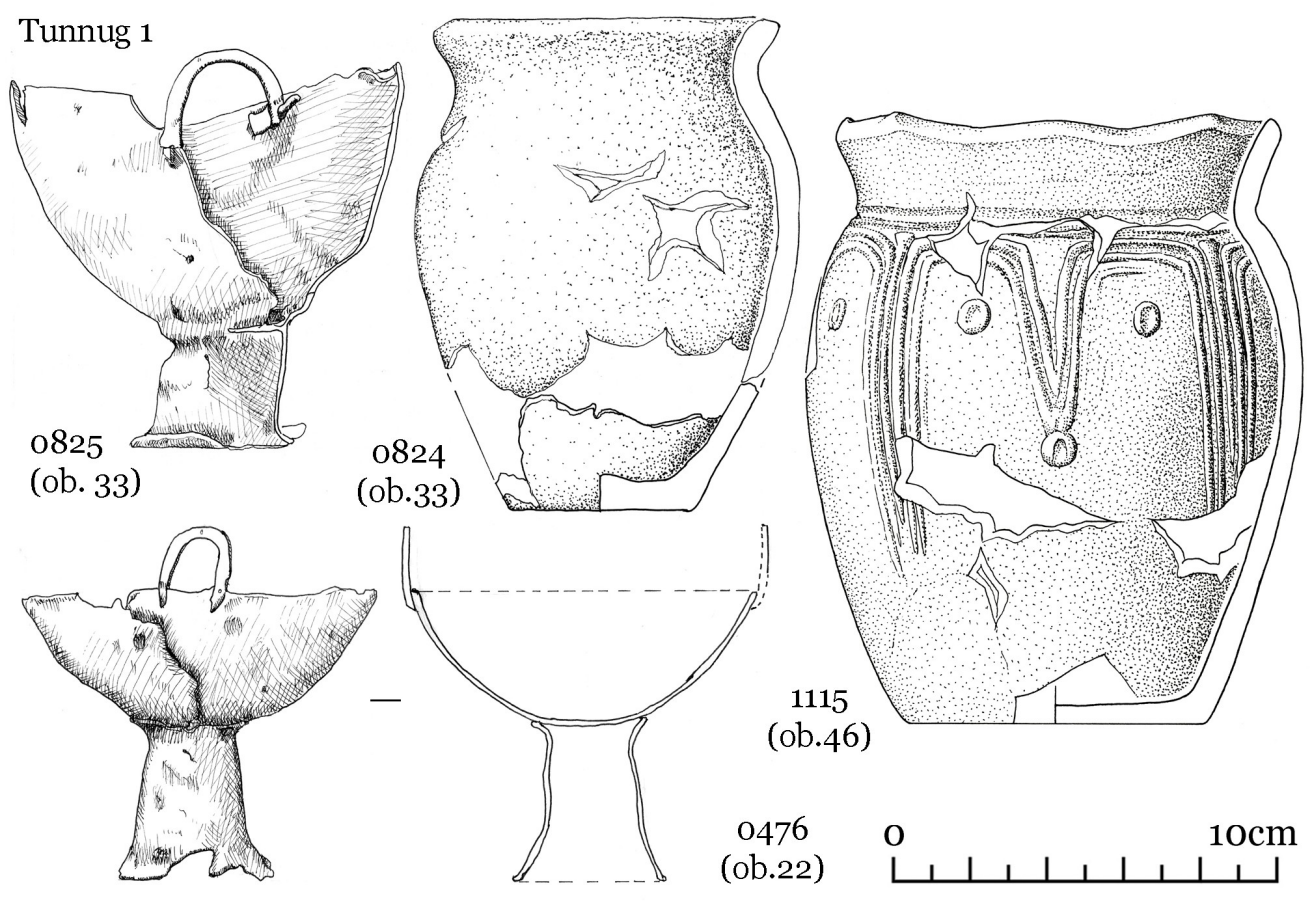
Vessels from objects 22, 33, 46. Vessels 476 and 825 are made from iron; 824 and 1115 are ceramic vessels.

**Fig 10:**
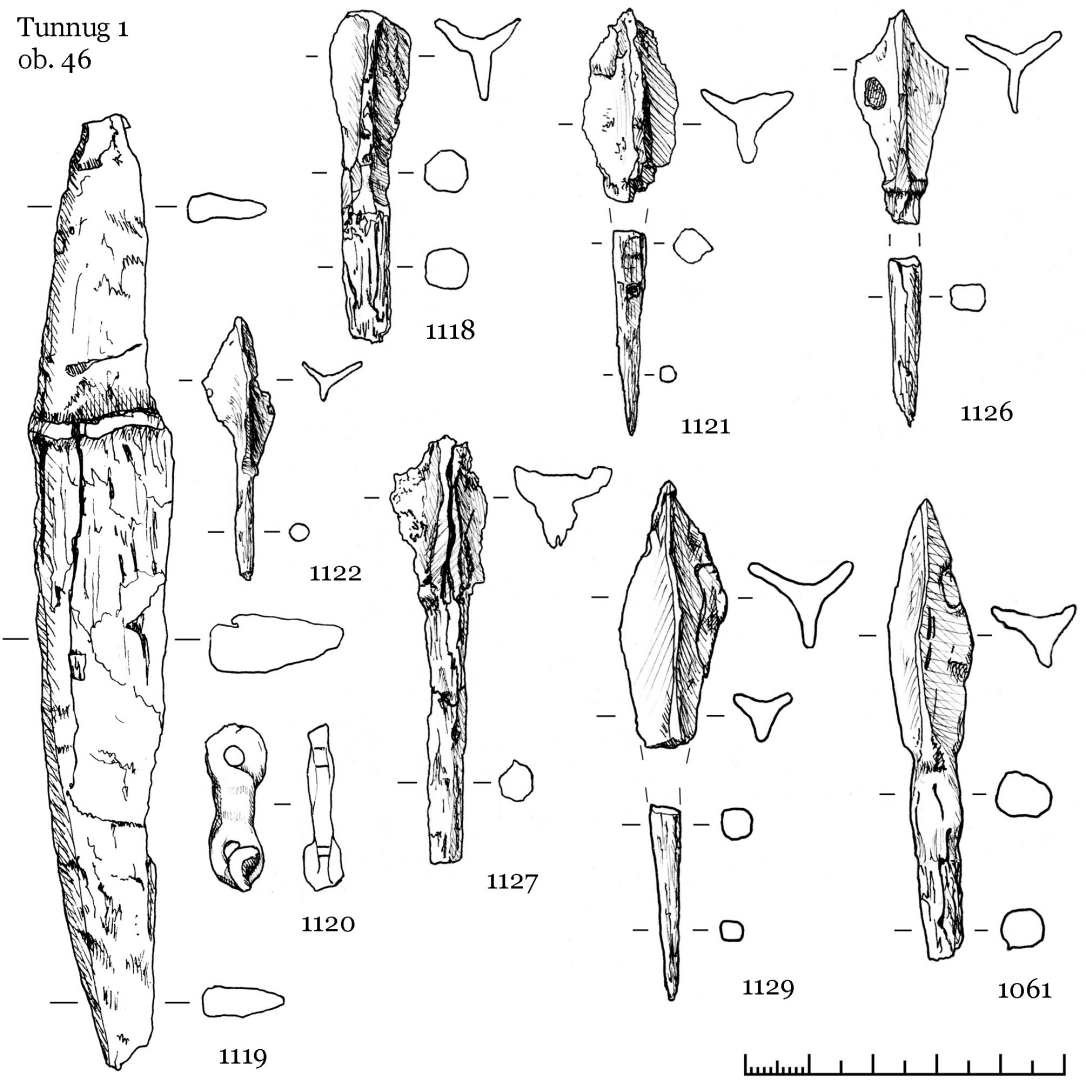
Iron finds from object 46. 1119—knife; 1118, 1121–1122, 1126–1127, 1129—arrowheads; 1120—item with unknown purpose.

Grave goods include a ceramic vessel with arched ornaments placed near the head of the deceased (Fig 9, 1115), an iron knife and a buckle near the abdominal area, as well as six iron petiolate three-lobed arrowheads (most likely the remains of a quiver interred with the individual) near the left hand and one arrowhead near the head (Fig 10, 1118, 1121, 1122, 1126, 1127, 1129).

#### 3.3.2 Object 33

**Fig 11:**
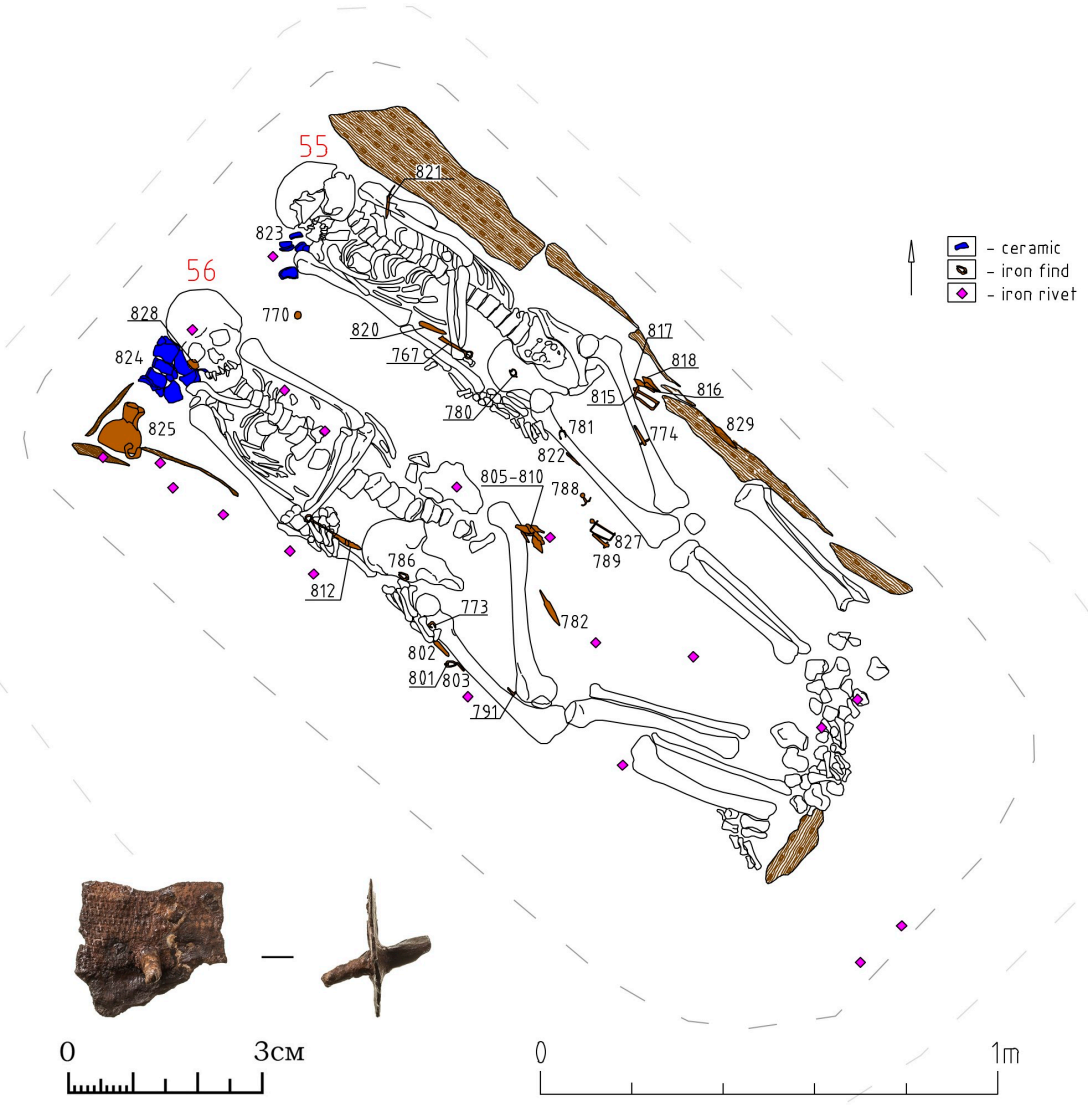
Object 33. Planigraphic view. Skeletons 55 and 56 buried in separate coffins. Iron rivets are depicted as purple diamonds, ceramics are marked in blue, iron finds are depicted in brown with the respective find number. The iron rivets display textile imprints on the corroded underside.

Object 33 is a burial pit covered by an accumulation of stones measuring 2.0 x 2.5m (Fig 11). The depth of the burial pit measured 1.1m from the ancient surface. The burial includes two inhumations buried in separate wooden coffins. This is suggested by the iron rivets only being present over one of the skeletons and spaced out regularly over the entire length of the body (Fig 11). The partial overlapping of the foot bones is probably due to the decomposition of the wood containers followed by frost movements at the site, which caused the displacement of individual 55. The finds included, among other items, more than 20 rivets (Fig 11). These are known to be used to fix textiles to the coffin covers, a custom widely spread since the Xiongnu period [38]. Judging by their location, only individual 56 was buried in a coffin with a textile cover. Textile imprints on the underside of the rivets during restorations confirmed this. Both individuals are male, 30–40 years old, and placed supine and extended with their heads to the northwest.

**Fig 12:**
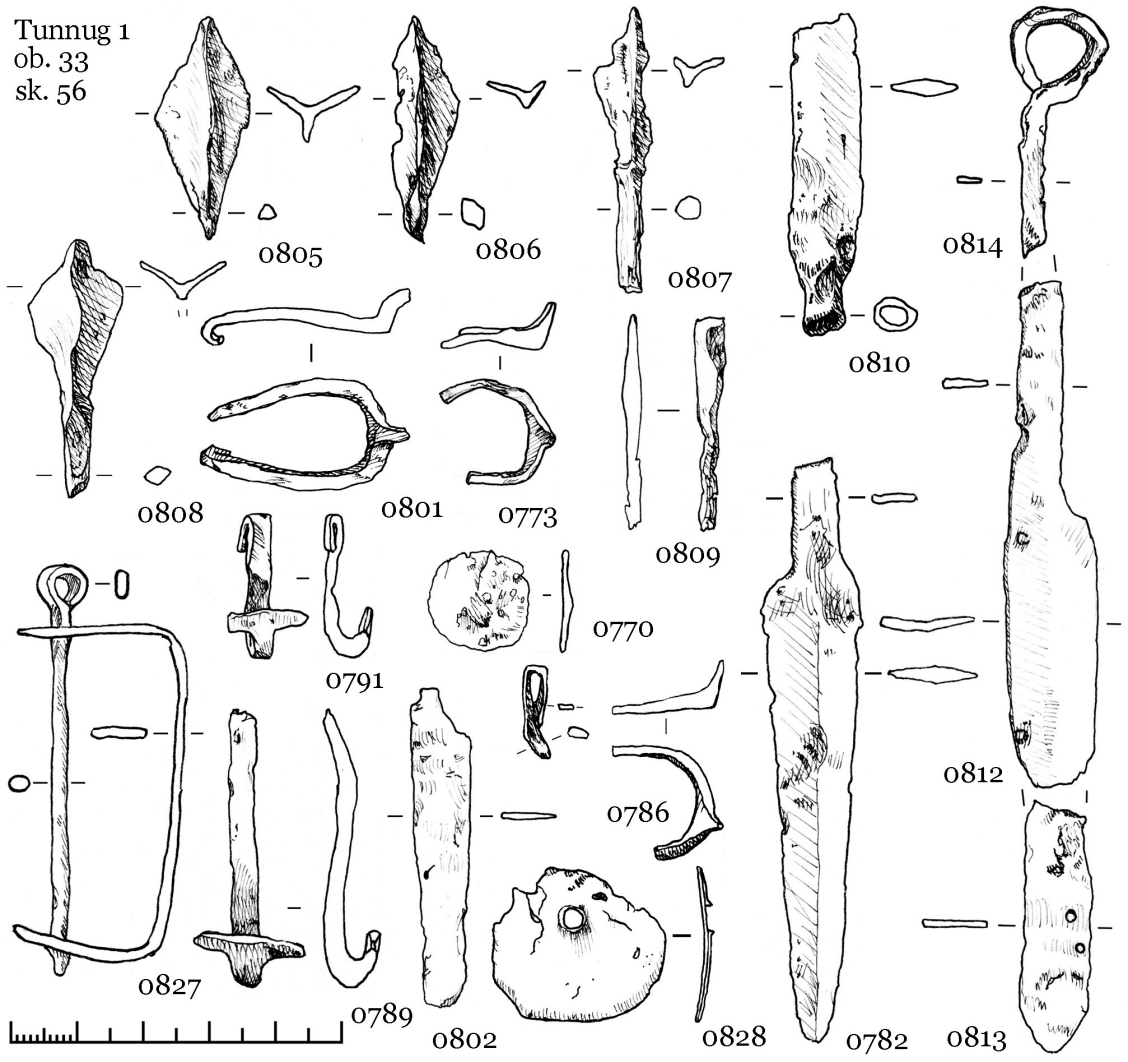
Object 33, inventory of skeleton 56.

A brace-shaped item (Fig 12, 0827) and a quiver hook (Fig 12, 0789) most likely belongs to the quiver of skeleton 56. Iron quiver hooks with a transverse bar are a common item in burials of the Kokel culture and among other, synchronous cultures from adjacent regions. However, brace-shaped items are quite rare. At date, we know only one other find in a burial from the Bulan-Koba culture in Altai [39: 321], where such an item was also found next to a quiver hook with a transverse bar. The functional purpose of these artefacts is not yet clarified, but it appears to be part of a quiver. The further inventory of skeleton 56 (Fig 12) consists of six iron arrowheads in one cluster, iron and ceramic vessels on the right near the head, an iron knife with a ring pommel at the right hand, knives near the legs, two buckles near the belt, a hook of a smaller size near the knees, a round iron plate with a hole near the skull.

**Fig 13:**
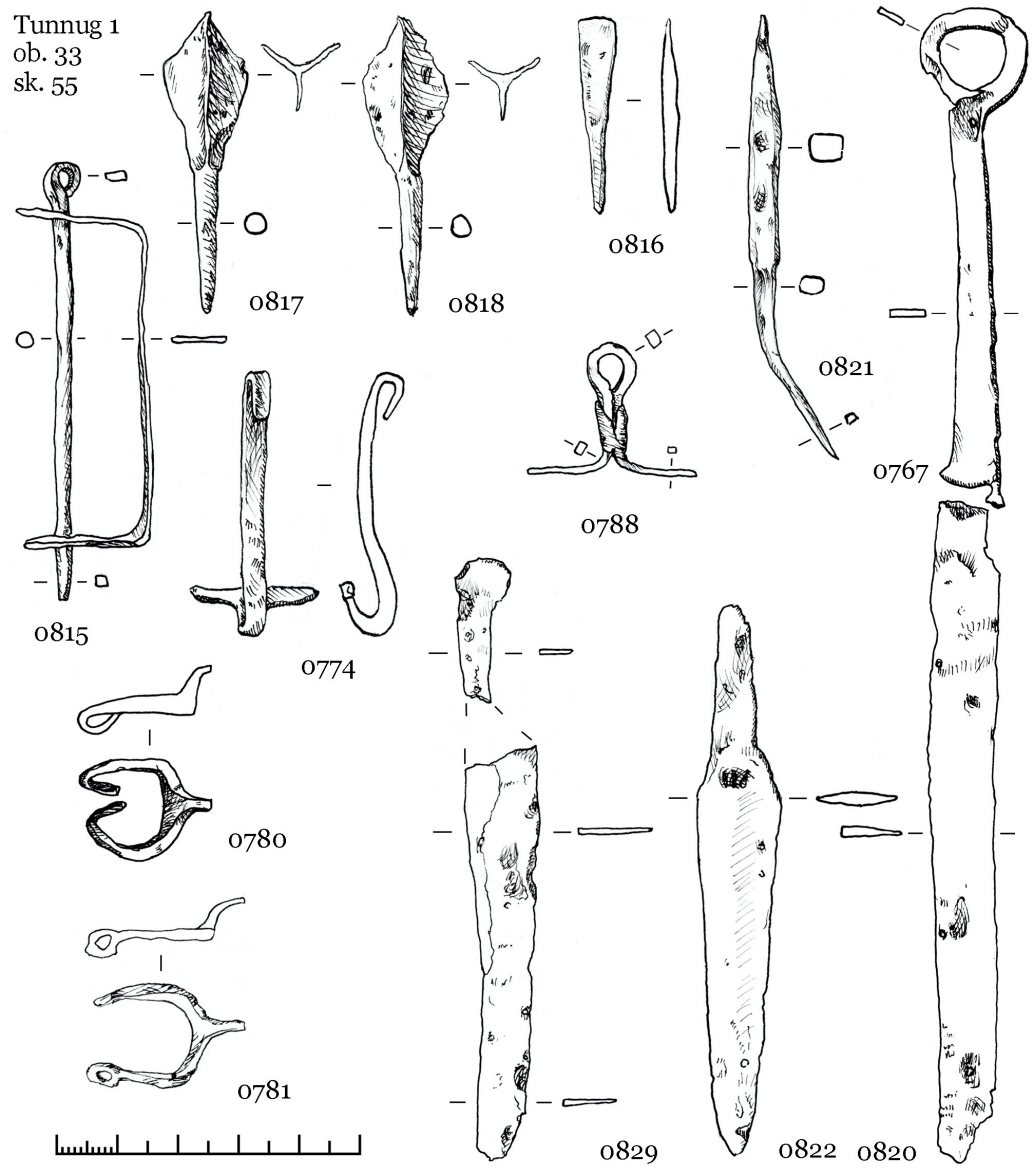
Object 33, inventory of skeleton 55.

The inventory of skeleton 55 (Fig 13) consists of three iron arrowheads in one cluster and one in the ribs (Fig 13: 0821), a brace-shaped iron item and a quiver hook near the arrowheads, two buckles, three iron knives, several fragments of ceramics on the right near the head (an incomplete vessel similar to Fig 9: 0824). Two knives are located on opposite sides of the legs, the third one with a ring pommel was found under the left hand. A small pair of iron buckles were found the first time in Kokel burials (Fig 13, 780; 781; Fig 12, 773; 801). They might be a derivative of the larger iron buckles with fixed tongue (cf. 39: Fig 57, 62, 100), which were widespread since the Xiongnu period.

#### 3.3.3 Object 22

**Fig 14:**
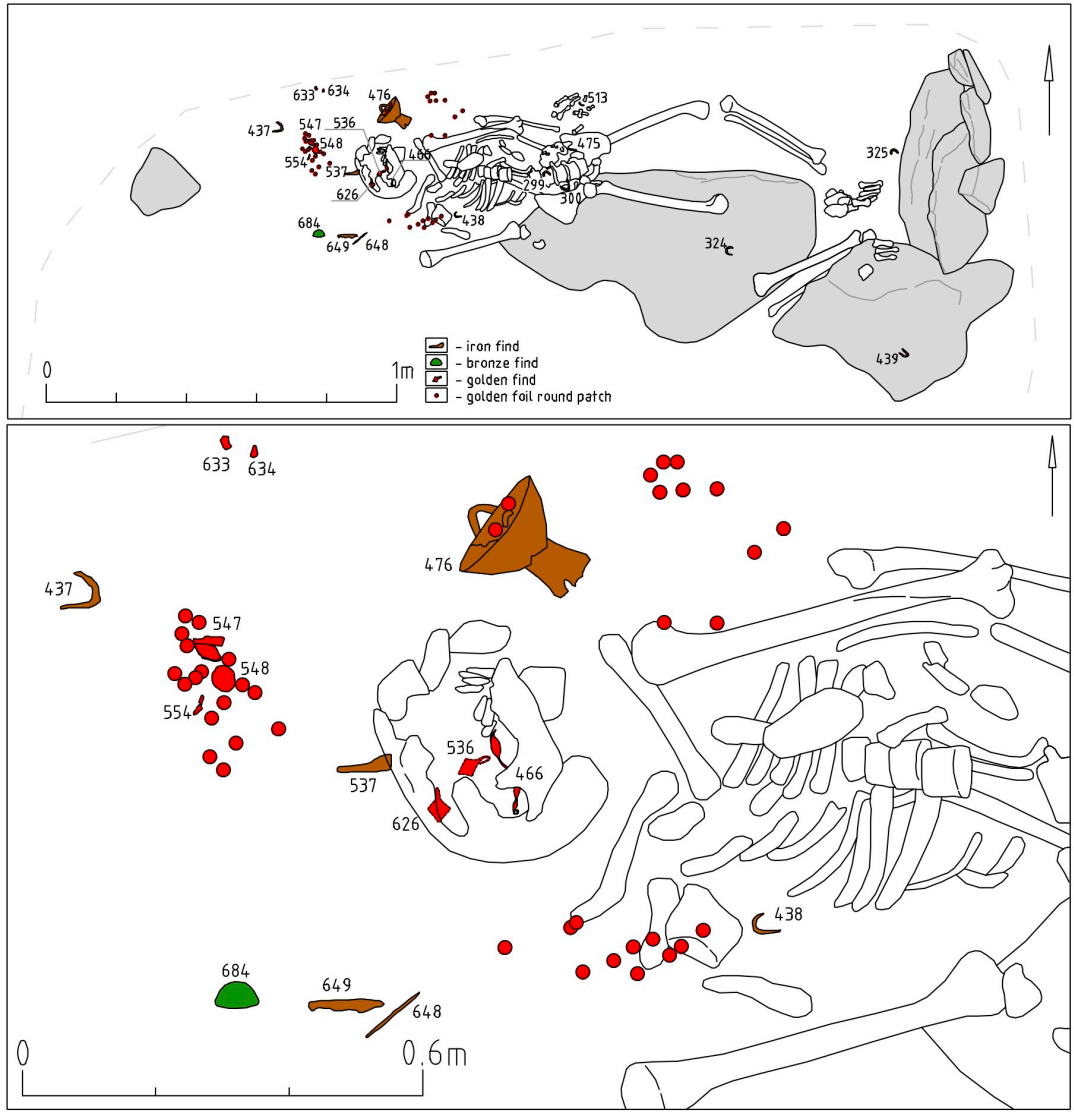
**a)** Planigraphic view of object 22. A large number of gold items was recovered from this burial. **b)** A close-up view of the head and surrounding gold items likely belonging to a headdress.

**Fig 15:**
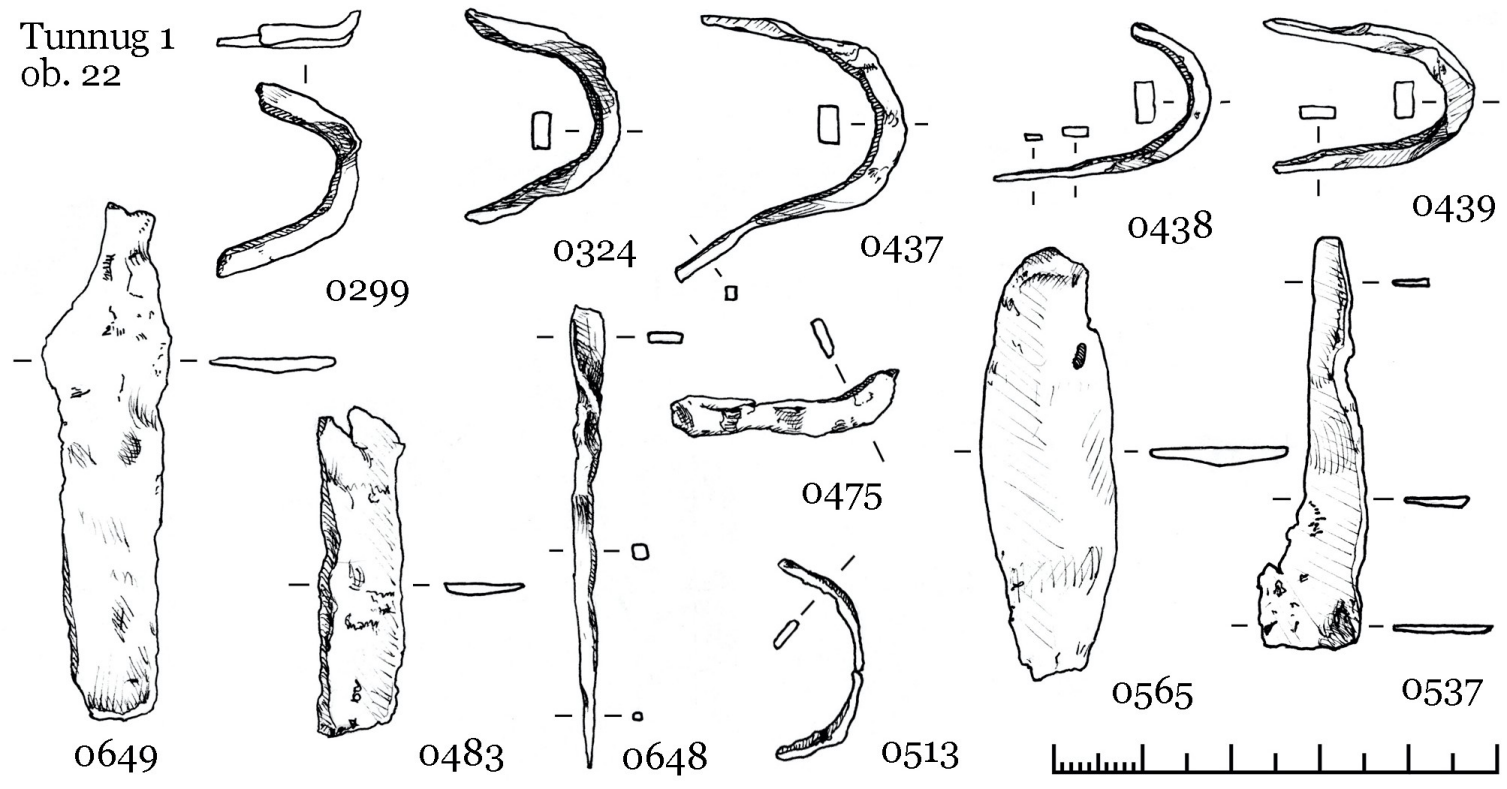
Fragmented iron finds from object 22.

Object 22 was disturbed by cryogenic processes, which caused the displacement of the skeletal remains. Traces of wood suggest the original presence of a wooden coffin. The inhumation sk. 39 is an adult female aged 25–30 years, she was interred in an elongated position with her head to the west (Fig 14). Her grave good inventory includes 82 items, 65 of which are made from gold. Beside the gold items, the rest of the funerary inventory is poorly preserved and fragmented (Fig 15). A miniaturized iron cauldron vessel was documented near the head on the left (Fig 14: 0476), fragments of two iron knives were found and two more fragments were discovered during sieving and washing the spoil heap. The rest of the iron items are fragmented but based on the position might be poorly preserved buckles.

**Fig 16:**
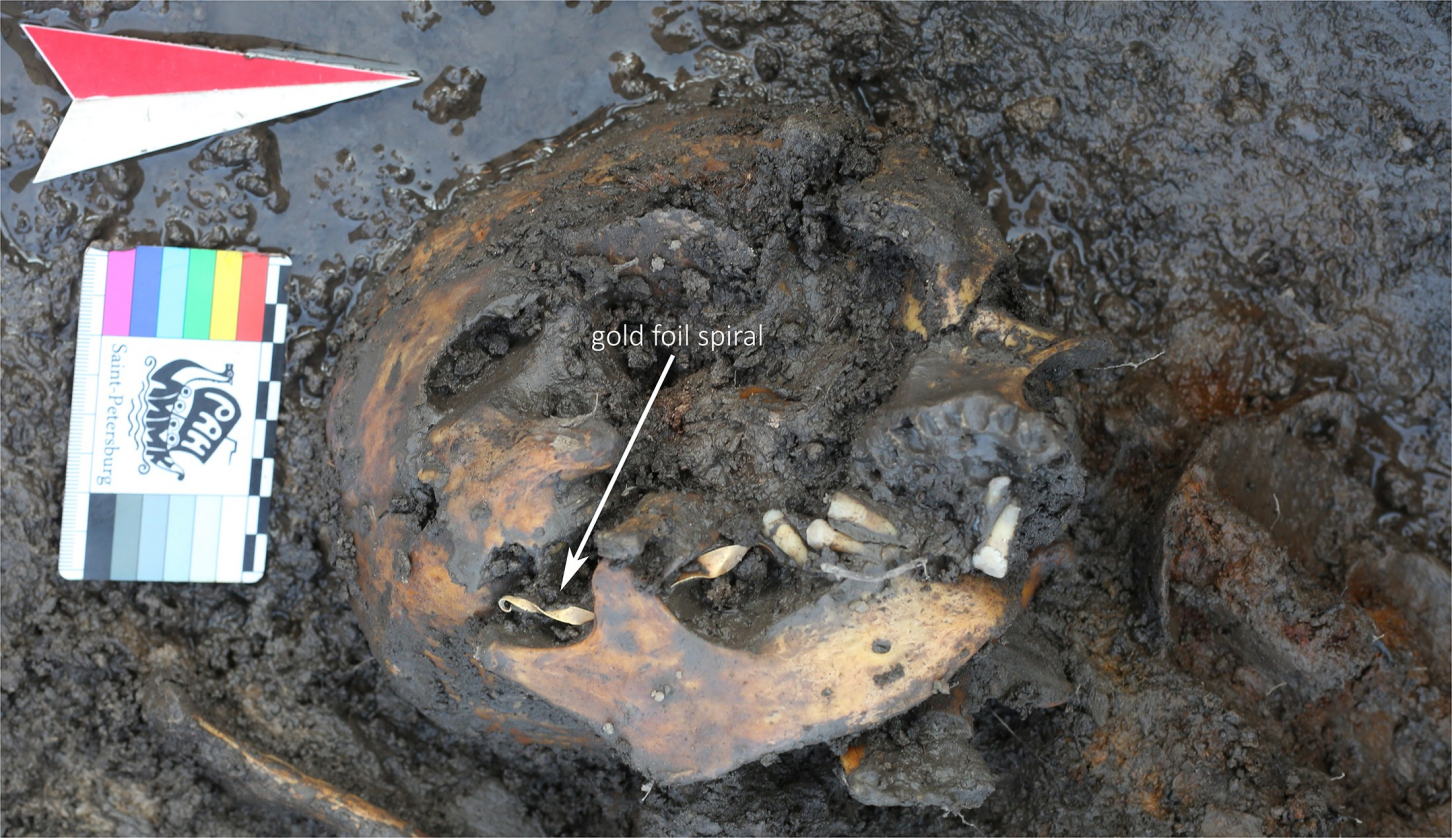
Gold foil spiral between the jaws of skeleton 39, object 22.

Fig 14b shows the upper part of the burial. We found a gold foil spiral between the mandible and maxilla of the deceased (Figs 14b and 16). Such finds are rare, although some were documented at the Kokel burial site [40: 170]. They appear to be present in both female and male burials. Originally referred to as “pectorals”, it is now clear that when found in context, they appear close to the skull. It could be that their intended placement is indeed in-between the jaws of the deceased, although further contexts have to be found where such a situation can be properly documented. Due to the taphonomic processes at the site, the placement of the gold spiral between the jaws is not without doubt. Another option would be the function as a chin strap which is known in different forms in the eastern steppes at the time [41]. The gold foil spirals seem to have had a specific function in the funerary ritual process rather than being items of everyday adornment.

**Fig 17:**
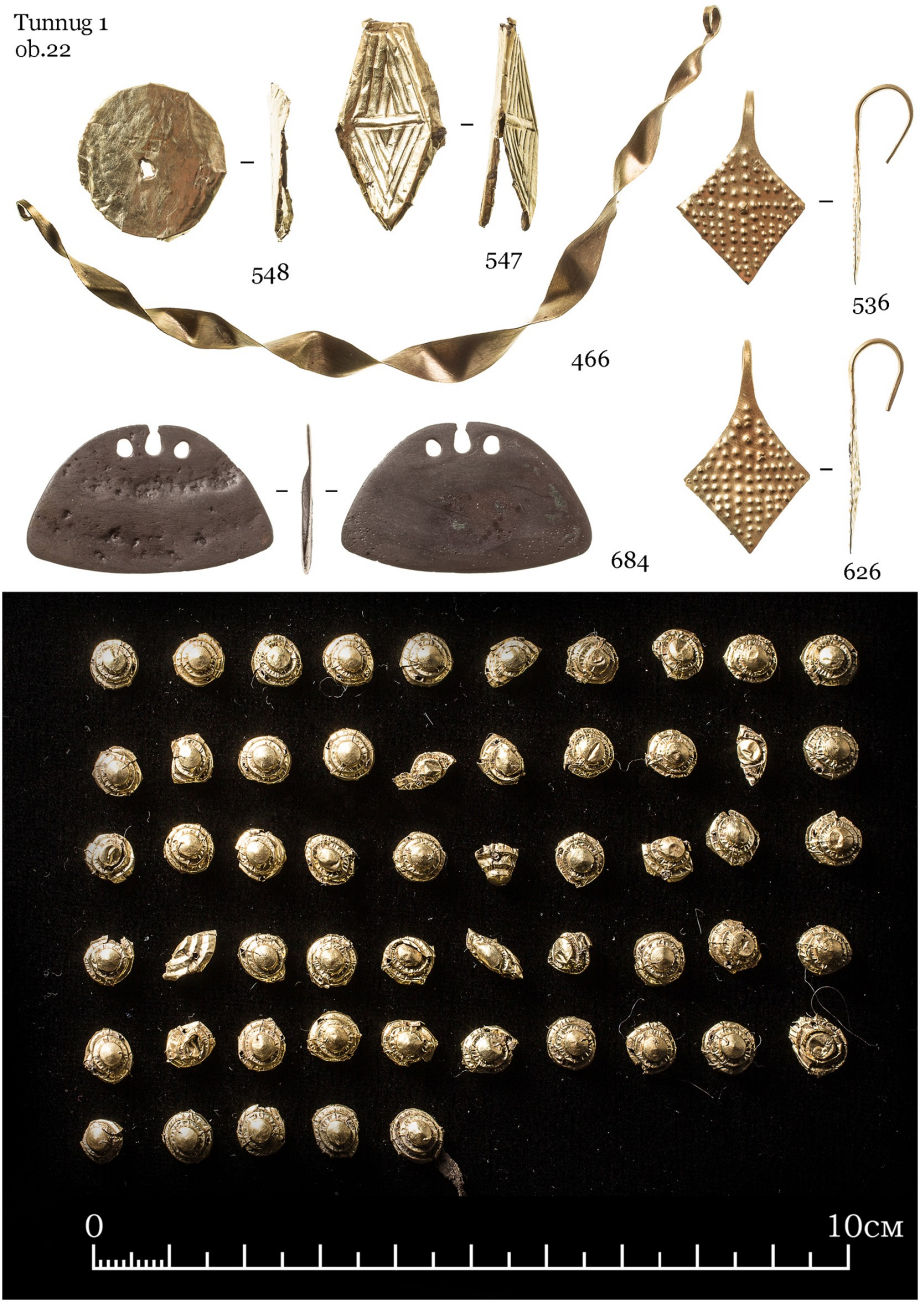
Bronze pendant (684) and gold foil adornments of the dead. Gold foil items likely belonging to an elaborate headdress.

Lozenge-shaped earrings (Fig 17: 536, 626) have no exact analogies in the region, but they are technologically similar to earrings found at the Kokel site [42: 1*74*] and sometimes excavated in burials of the Bulan-Koba culture of Altai [43: 114–115]. Smaller gold items are located in three clusters around the head (Figs 14 and 17) which suggests that they likely were part of a headdress. The only bronze artefact is a small pendant (Fig 17: 684), and likely was not a cast item but a reused fragment of a Chinese bronze mirror. Fragments of Han Chinese mirrors are known from the Kokel burial site, and the sizes of these fragments can be very small [44: 230]. The reconstructed diameter of the mirror (based on the curvature of the edge) is about 9 cm, which would be consistent with the size of some Han era mirrors.

### 3.4 Radiocarbon dates

Until recently, only a few radiocarbon dates were available for Kokel sites. A small series of samples was measured for the Katylyg 5 fortified settlement (Table 1, cf. [16]), with almost all estimates falling between the 2^nd^–4^th^ centuries CE (2σ). A larger series of samples from the Tunnug 1 site reflects a similar time range (Table 2). While no new radiocarbon dates other than the already published ones are reported here, we collected all available radiocarbon dates for Kokel contexts and recalibrated them. Radiocarbon ages were translated into calendar ages with OxCal 4.3 [45] using the IntCal20 calibration curve [46].

**Table 1:** Uncalibrated and calibrated radiocarbon dates for features from Katylyg 5 fortified settlement (see also [16]), measurements were carried out at the Laboratory of Archaeological Technology at the Institute for the History of Material Culture, Saint-Petersburg, Russia.

*Lab Code*	*Feature N*	*14C age (BP)*	*Calendar age (2σ)*	*Material*
*LE-10937*	*21*	*1730±20*	*CE 250–404*	*charcoal*
*LE-10938*	*716*	*1760±20*	*CE 238–352*	*charcoal*
*LE-10940*	*719*	*1720±20*	*CE 254–406*	*charcoal*
*LE-10941*	*44*	*1700±25*	*CE 257–415*	*charcoal*
*LE-10942*	*150*	*1710±30*	*CE 252–416*	*charcoal*
*LE-10943*	*311*	*1770±30*	*CE 233–375*	*charcoal*
*LE-10944*	*482*	*1770±30*	*CE 223–375*	*charcoal*
*LE-10945*	*20*	*1820±45*	*CE 88–345*	*charcoal*

**Table 2:** Uncalibrated and calibrated radiocarbon dates for Kokel period human remains from Tunnug.

*Skeleton Nr*.	*Lab Code*	*Funerary structure (object)*	^*14*^*C age (BP)*	*C%*	*C*:*N*	*Calendar age (2σ)*
*1*	*BE-11033*.*1*.*1*	*5*	*1791±20*	*43*.*9*	*3*.*1*	*CE 218–330*
*3*	*BE-11034*.*1*.*1*	*n*.*a*.	*1773±20*	*45*.*5*	*3*.*2*	*CE 235–344*
*4*	*BE-11035*.*1*.*1*	*n*.*a*.	*1723±20*	*48*.*6*	*3*.*1*	*CE 252–407*
*7*	*BE-11036*.*1*.*1*	*5*	*1714±20*	*45*.*8*	*3*.*1*	*CE 255–409*
*10*	*BE-11037*.*1*.*1*	*n*.*a*.	*1753±20*	*42*.*6*	*3*.*1*	*CE 241–365*
*14*	*BE-11038*.*1*.*1*	*12*	*1740±20*	*42*.*3*	*3*.*1*	*CE 246–383*
*15*	*BE-11039*.*1*.*1*	*13*	*1781±20*	*48*.*2*	*3*.*1*	*CE 231–339*
*18*	*BE-11040*.*1*.*1*	*16*	*1628±20*	*41*.*7*	*3*.*1*	*CE 406–537*
*25*	*BE-12824*.*1*.*1*	*n*.*a*.	*1768±20*	*48*.*9*	*3*.*2*	*CE 237–346*
*33*	*BE-12822*.*1*.*1*	*18*	*1743±20*	*50*.*5*	*3*.*1*	*CE 245–380*
*46*	*BE-12823*.*1*.*1*	*24*	*1755±20*	*50*.*2*	*3*.*1*	*CE 241–362*
*55*	*BE-12821*.*1*.*1*	*33*	*1726±20*	*50*.*1*	*3*.*1*	*CE 251–405*
*57*	*BE-13065*.*1*.*1*	*34*	*1747±19*	*50*.*2*	*3*.*1*	*CE 245–376*
*58*	*BE-13066*.*1*.*1*	*34*	*1716±19*	*50*.*2*	*3*.*1*	*CE 255–408*
*59*	*BE-13067*.*1*.*1*	*34*	*1765±20*	*43*.*4*	*3*.*1*	*CE 237–348*
*60*	*BE-12826*.*1*.*2*	*34*	*1724±19*	*48*.*6*	*3*.*1*	*CE 252–406*
*68*	*BE-12825*.*1*.*1*	*42*	*1746±20*	*50*.*5*	*3*.*1*	*CE 245–376*
*94*	*BE-12827*.*1*.*1*	*17*	*1787±20*	*51*.*2*	*3*.*1*	*CE 224–335*
*95*	*BE-12828*.*1*.*1*	*17*	*1738±20*	*51*.*0*	*3*.*2*	*CE 247–401*

Measurements were carried out at the LARA laboratory at the Department of Chemistry and Biochemistry at the University of Bern.

## 4. Discussion

**Fig 18:**
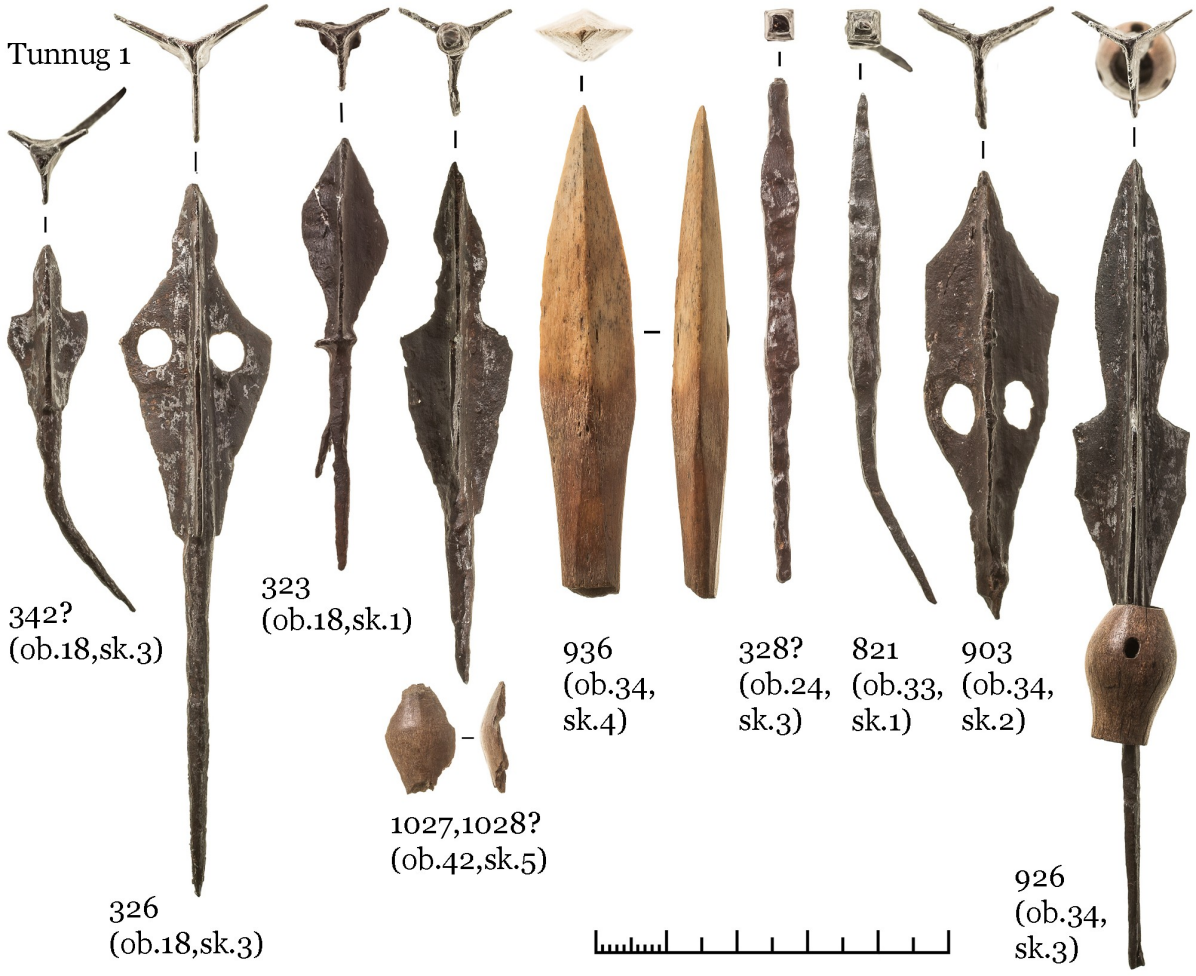
Arrowheads found in skeletal regions of individuals in Kokel burials at Tunnug.

The archaeological record of the first centuries CE in Tuva does not indicate steep social hierarchies or the presence of an elite. Burials remain relatively uniform and the variation in grave goods is limited. So far we do not know of any archaeological structures which appear to have been constructed for a specific person of high status. Some burials contain gold items (like object 22), but it seems likely that these have to be seen as personal adornment and less as a sign of status, since the overall setting of the funerary ritual does not show other adaptions. Individuals with gold items were buried with the same ceramics and iron items, in the same places within similarly sized pits. Elite burials are well known for the Early Iron Age, the Xiongnu period, and the Turkic Khaganates, but none of the funerary sites dating to the 2nd -4th centuries CE exhibit characteristics of elite funerary customs. The remarkable absence of elite burials could have reasons beyond a lack of control by a central authority, but drawing conclusions based on the absence of data is tenuous. We interpret the high proportion of violent deaths as a further indication of small-scale conflict. The numerous chop marks on some skeletons seem to indicate unorderly raids and the overall distribution of skeletal trauma shows that people fought both on foot and from horseback [36]. Frequent finds of arrowheads in skeletal regions add to these observations. Our excavation has yielded nine arrowheads from different burials which might have caused severe injuries (Fig 18). Interestingly, these arrowheads are often typologically different from the ones found in the quivers which were placed next to the deceased as grave goods [47]. The diversity of forms seen in the arrowheads recovered from skeletal regions is apparent. They do not show the standardization expected if the victims were shot by the projectiles of a well-organized military unit.

Other scholars have suggested larger scale violent conflict as an explanation for the presence of frequent skeletal trauma in Kokel sites. Savinov [1] considers the high proportion of violent deaths in Kokel burials as an indication of a possible Xianbei presence in Tuva, implying at least regional military conflicts in the framework of the Xiongnu Xianbei Chinese wars of the 1st century BCE– 1st century CE. The recent radiocarbon dates make this idea improbable, since there is so far no site of Kokel material culture which has been securely dated to this timeframe.

Preliminary zooarchaeological evidence from Katylyg 5 shows that the economy was based on transhumant pastoralism, likely without supraregional mobility [17: 140] The archaeological record is dominated by local products in particular iron items and ceramics. Katylyg 5 shows signs of local metal production [17: 142]. Sourcing analyses of the clay used for the vessels in the burials are on-going. Archaeological items that would allow for an interpretation as the result of non-local contacts are few if any. Even the rare bronze pendant (Fig 17, 684) possibly belonging to a Han Dynasty mirror does not have to be a direct import.

The excavations at Tunnug revealed items not previously known in this form from other Kokel sites. Iron buckles (Fig 13: 780, 781) and unusual iron elements for fastening quivers (Fig 12: 827) might be further arguments for local traditions of small-scale tribal groups. A preliminary chronological subdivision of Kokel burials into two groups has been suggested by Savinov [1:14]. The subgroups have not been clearly defined but it is possible to list the main features. In the “Central Tuvan” variant (the early stage) burials usually contain little inventory, arrowheads are three-lobed and “over vessel mounds” are widespread. For the “Western Tuvan” variant (the later stage), cauldron-like vessels, miniaturized model of items, knives with ringed pommels, and multiple burials in the same pit are common. The materials we presented can be categorized as belonging to either one of these two groups. Object 46 would be categorized into the “Central Tuvan” variant while objects 33 and 22 would be considered belonging to the “Western Tuvan” variant. Both subcategories of Kokel burials are present at Tunnug. However, the chronological distinction between the subgroups seems to be reversed here. Burials belonging to the “Western Tuvan” variant are located in the center area while “Central Tuvan” style burials are stratigraphically later. It therefore remains to be determined whether these variants are the expression of local, social, cultural or chronological differences. This issue requires a separate study, which will be carried out in the near future.

With the decline of the Xiongnu Empire, social groups in Tuva likely return to small-scale tribal organization and long-distance connections are no longer maintained. The archaeological record of the time lacks prestige goods and items which can be identified as imports. The collapse of supraregional contacts and power structures of a “super-complex chiefdom” [48] likely led to a significant reduction in trade, and possibly in economic prosperity. An increasing compartmentalization and localization of material assemblages mirrors the decline of central power.

The social and political history of the post-Xiongnu time is only understandable in the most general outline. While written sources paint a relatively detailed picture of the Eastern steppes during the time of conflict between the Han Dynasty and the Xiongnu, the time period during which the Kokel material culture appears has to be treated as essentially Prehistoric. We deliberately use the term “post-Xiongnu time”, because despite a clear chronological separation of the two material cultures, Xiongnu material culture still serves as a distant precursor. From an archaeological point of view, the Kokel archaeological culture is more closely related to the Xiongnu culture than to the later Turkic material culture. The fact that it was considered a variation of Xiongnu material culture by earlier scholars [2, 3] aides in this categorization. Even for the time of the Xiongnu Empire, the Chinese sources remain largely silent regarding the distant reaches of the nomadic empire. The reconstruction of social and political history in the belt of the steppes remains controversial [48]. Tuva seems to have been integrated into the Xiongnu Empire at times or at least would have incorporated customs which were typical for a larger material culture complex. The few sites in Tuva which show an affiliation with Xiongnu material culture, however, also exhibit traits of local cultural influences [7].

## 5. Conclusion

Through increasing data availability the view on the archaeological materials and associated socio-economic circumstances in Tuva in the first centuries CE have changed considerably. The originally proposed explanation for the change in patterns of material culture, namely a superimposition of Xiongnu material culture on a local post-Scythian substrate, is no longer tenable. Radiocarbon dates have allowed to confirm the proposed reduction of the wide time span from seven centuries to a relatively short period between the first and fourth century CE, thus revealing a significant chronological gap in the Late Prehistory of Tuva. The societies associated with the Kokel material culture are likely small-scale tribal organizations centered around extended families. No pronounced social hierarchy can be observed based on the funerary ritual patterns. A lack of imported goods and localized archaeological materials speak towards a limited geographical extent of these groups and a time in which local subsistence economy and power structures functioned largely independently from long-distance trade and prestige goods. Competition over local resources was fierce. Violent conflicts and the presence of skeletal trauma speak towards raiding activities and the crucial role of small-scale warfare among tribal groups. The situation changes dramatically with the expansion of the Turks and the rise of the First Turkic Khaganate in the 6th century CE, when bronze and prestige items reappear, and a supraregional material culture, as well as long-distance contacts emerge.
